# The influence of subanaesthetic ketamine on regional cerebral blood flow in healthy dogs measured with ^99m^Tc-HMPAO SPECT

**DOI:** 10.1371/journal.pone.0209316

**Published:** 2018-12-18

**Authors:** Lise Vlerick, Kathelijne Peremans, Robrecht Dockx, Kurt Audenaert, Chris Baeken, Bart De Spiegeleer, Jimmy Saunders, Ingeborgh Polis

**Affiliations:** 1 Department of Small Animal, Faculty of Veterinary Medicine, Ghent University, Merelbeke, East Flanders, Belgium; 2 Department of Veterinary Medical Imaging and Small Animal Orthopaedics, Faculty of Veterinary Medicine, Ghent University, Merelbeke, East Flanders, Belgium; 3 Ghent Experimental Psychiatry (GHEP) lab, Department of Psychiatry and Medical Psychology, Ghent University, Ghent, East Flanders, Belgium; 4 Drug Quality and Registration (DruQuaR) group, Faculty of Pharmaceutical Sciences, Ghent University, Ghent, East Flanders, Belgium; Columbia University, UNITED STATES

## Abstract

Subanaesthetic ketamine has recently been proven to be a highly effective and fast acting alternative treatment for several psychiatric disorders. The mechanisms responsible for ketamine’s antidepressant effects remain unclear, but a possible explanation could be that ketamine interacts with regional cerebral blood flow (rCBF). Therefore, the effects of two subanaesthetic ketamine doses on rCBF were evaluated. Twelve dogs were randomly assigned to one of the three treatment conditions (condition saline, condition 0.5 mg/kg ketamine or condition 2 mg/kg ketamine) and received in total five saline or ketamine infusions, with one week interval. Single Photon Emission Computed Tomography (SPECT) scans with the radiotracer ^99m^Tc-hexamethylpropylene amine oxime were performed before the start of the infusions (baseline) and 24 hours after the first (single) and last (multiple) infusion. After a wash out period of 3 months, the animals were again assigned to one of the three treatment conditions described above and the infusion/scan protocol was repeated. During the infusions, cardiovascular parameters were evaluated every ten minutes. A one-way repeated measure ANOVA was set up to assess perfusion index for each ketamine dose for the left frontal cortex (alpha = 0.05). The remaining 11 brain regions were post hoc assessed. Perfusion index was significantly increased in the left frontal cortex and in the thalamus 24 hours after single and multiple ketamine infusions compared to baseline in the 2 mg/kg condition. No clinically relevant cardiovascular effects were observed during the ketamine infusions. This study shows that subanaesthetic ketamine can increase neuronal perfusion and therefore alter neuronal function in brain regions involved in depression and anxiety disorders. These perfusion increases may possibly contribute to ketamine’s beneficial effects in these psychiatric disorders.

## Introduction

Ketamine, a dissociative anaesthetic, is routinely used in human and veterinary medicine [1]. It mainly acts as a non-competitive N-methyl-D-aspartate receptor antagonist but in combination with norketamine, its active metabolite, targets other receptors. These include the dopamine D_2_ receptor and opioid receptors [2] and the 5-HT2_A_ receptor [3,4].

In human medicine, the current clinical value of ketamine, besides its well-known anaesthetic properties, lies mainly in psychiatry and pain management [5]. In psychiatry, subanaesthetic dosed ketamine has already been shown to be a rapid and effective treatment for bipolar disorder, suicidal ideation and major depressive disorder (MDD) [6–11]. Ketamine has also demonstrated efficacy for Post-Traumatic Stress Disorder (PTSD) [12].

A classic ketamine treatment scheme for MDD consists of intravenous administration of 0.5 mg/kg ketamine infused over 40 minutes [6–9]. Importantly, the effects of a single ketamine infusion last long after the drug is metabolized (beyond its 3h half-life in humans) with sustained responses for up to one week on average [6,8,9]. Moreover, repeated ketamine infusions may prolong the durability of the antidepressant response and are associated with higher response and remission rates compared with a single infusion [13–15].

In veterinary medicine ketamine is routinely used both in small animals and in large animals, mainly for its sedative, anaesthetic, and analgesic properties. In subanaesthetic doses in dogs, ketamine is used in combination with other analgesics to augment analgesia in the peri- and postoperative period [16,17]. Ketamine has been proven to be particularly useful for the management of neuropathic pain [18]. When used for pain management, low doses of ketamine are administered by constant rate infusion, usually as an adjunct to other analgesics such as morphine or fentanyl [16,17].

Although there has been increasing interest in the use of ketamine in human psychiatry, no consensus has been reached on the neurobiological mechanisms accounting for ketamine’s effects on patients with mood and anxiety disorders [3,19]. An animal model may be helpful in unravelling its mechanism of action. The dog is considered to be a valuable animal model for several human psychiatric conditions [20,21]. Not only because dogs exhibit naturally occurring behavioural disorders that are homologues to certain human psychiatric conditions [20], but also since several functional imaging studies demonstrated similar changes in neuronal activity and cerebral blood flow. These changes were reported both in dogs [22–25] and in humans suffering from behavioural disorders such as anxiety disorders, impulsive aggression, and compulsive disorders [26–28]. The left frontal cortex is strongly implicated in the pathophysiology of these behavioural disorders, both in humans and dogs, and altered perfusion of this brain region is a common characteristic in these diseases [26,27,29–31]. Additionally, various studies in human and veterinary medicine showed altered perfusion in the (left) frontal cortex, following administration of subanaesthetic ketamine [32–37]. All these studies focused on regional cerebral blood flow (rCBF) evaluation during or shortly after a single ketamine infusion. Since responses after a single ketamine infusion can last up to one week or longer, it would be interesting to evaluate rCBF at a later stage (24h) following the termination of the infusion and after multiple ketamine infusions. As such, more insights in the neurobiological effects and optimization of the ketamine dosing protocol could lead to a more successful therapy for patients with MDD and/or anxiety disorders.

Therefore, the first objective of the current study was to evaluate the effects of two subanaesthetic ketamine doses on left frontal cortical perfusion in healthy dogs, 24 hours after single and multiple ketamine infusions, placebo controlled, measured with Single Photon Emission Computed Tomography (SPECT). A second objective was to identify possible adverse effects of the two ketamine doses on cardiovascular parameters and on behaviour.

## Materials and methods

### Animals

Twelve neutered adult dogs (10 females, 2 males; Beagles; age 2.7 ± 0.3 years; weight 11.7 ± 0.8 kg) were included in this study. The animals had no history of neurological or behavioural disorders and were classified as healthy based on general clinical examination. This study was approved by the local Ethical Committee of the Faculty of Veterinary Medicine, Ghent University (EC 2015_130 and EC 2016_60) and all procedures were performed according to good animal practice. Welfare of the animals was respected at each time and great care was taken to avoid stress and anxiety. No animals were sacrificed. The dogs were provided by the small animal department and the department of veterinary medical imaging and small animal orthopedics of the faculty of veterinary medicine. The dogs were socially-housed in small groups (2 to 8 dogs) on an internal surface of 15 m^2^ with permanent access to an outside area of 15 m^2^. The bedding material in the inner part consisted of wood shavings. Feeding toys, such as Kongs were provided on a regular basis. Twice a day, the animals were allowed to run and play outside in an enclosed play area, enriched with climbing platforms, hiding places and small bushes. In addition, the dogs were regularly walked by students of the faculty of veterinary medicine.

### Study design

**Fig 1:**
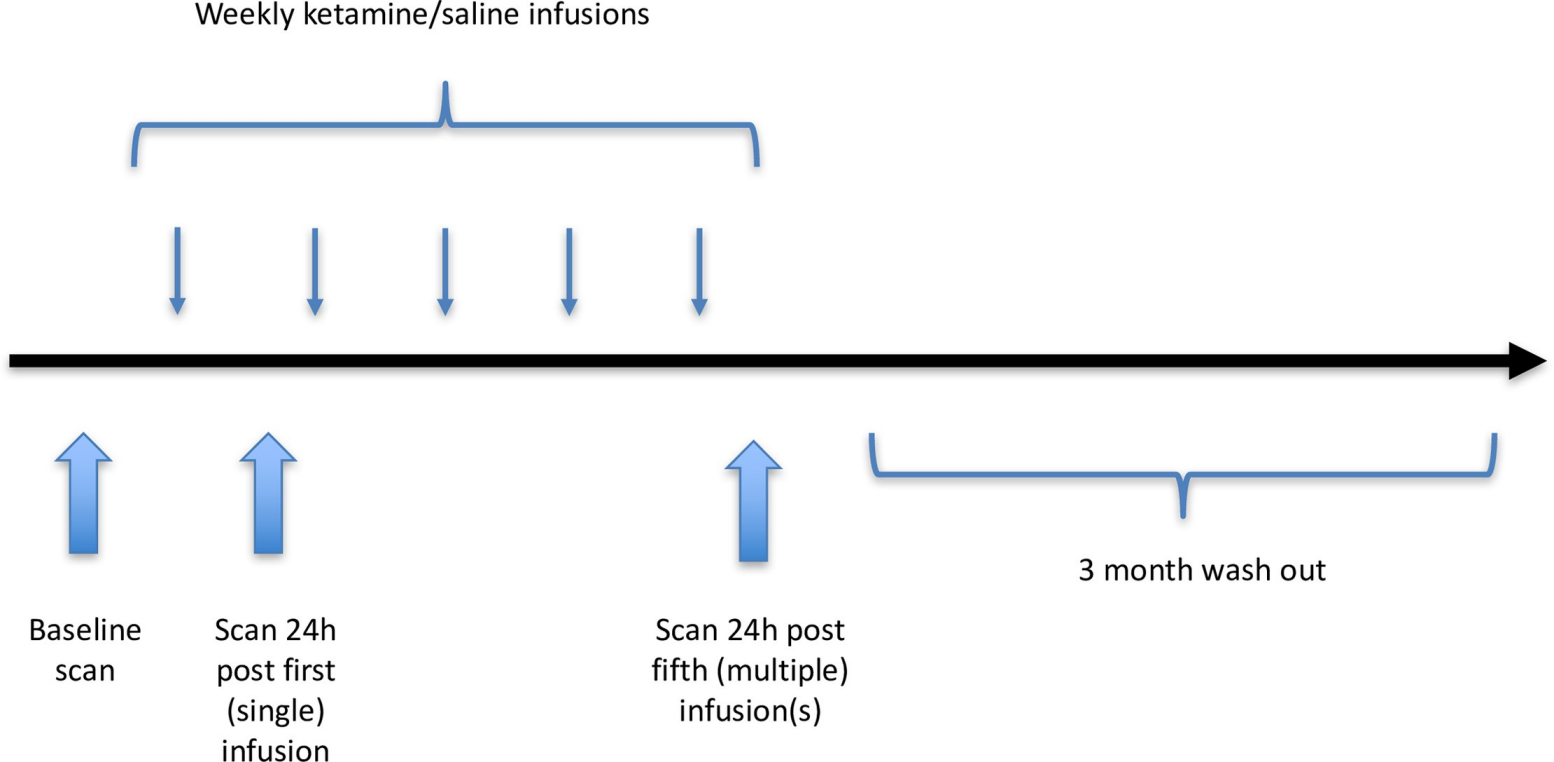
Schematic overview of the study design. Each animal underwent the infusion/scan protocol twice, although different treatment, with a wash out period of three months in between the two sessions.

The effects of two subanaesthetic ketamine doses (0.5 mg/kg and 2 mg/kg, Nimatek, Eurovet Animal Health B.V, Bladel, Nederland) on rCBF were examined, using Single Photon Emission Computed Tomography (SPECT) with the radiotracer ^99m^Tc-hexamethylpropylene amine oxime. A sample size calculation was performed based on a prediction linear mixed model with a delta (predicated difference) equal to 0.05 and a power of 0.80. This provided a sample size of 7.48 animals per group. Consequently, a sample size of 8 animals per group was chosen. Therefore, in the first part of the study, twelve dogs were randomly divided into three groups, with one group receiving ketamine at a dose of 0.5 mg/kg (condition 0.5 mg/kg), one group receiving ketamine at 2 mg/kg (condition 2 mg/kg) and one group receiving saline (condition saline). Each animal received in total five ketamine or saline infusions, with one week interval. SPECT scans were performed at three time points: one to two weeks prior to the infusions (baseline scan) and 24 hours after the first (single) and last (fifth/multiple) infusion (Fig 1). In all groups, the total dose of ketamine or saline was administered intravenously over 40-minutes. The dosages used are in the order of what it is described in human psychiatry, taken into account differences in pharmacokinetics between species [6–9]. Prior to the infusions, the dogs were sedated intramuscularly in order to avoid side effects due to the ketamine administration. A wash out period of 3 months was respected before the second part of the study was initiated. In this second part of the study, the animals were reused and again assigned to one of the three treatment conditions described above. Once again, each dog received five weekly ketamine or saline infusions, preceded by a baseline SPECT scan and followed by a SPECT scan after the first and fifth infusion. In summary, each animal underwent the infusion/scan protocol twice, although different treatment, with a wash-out period of three months in between the two sessions. Consequently, all dogs participated in two of the three treatment conditions and eight dogs were included in each treatment condition.

A validated questionnaire (Canine Behavioural Assessment and Research Questionnaire, C-BARQ) was used to score each dog’s behaviour before and after the ketamine or saline administrations [38]. As not all categories of the questionnaire were relevant for this study, only items regarding aggression, fear and anxiety were evaluated. The principal animal caretaker completed the questionnaires. Responses were evaluated in terms of intensity of the behaviours (aggression, fear and anxiety), using a 5-point qualitative rating scale (0 = no signs of the behaviour, 1 to 3 = mild to moderate signs of the behaviour, and 4 = severe signs of the behaviour).

### Ketamine administration protocol

Following intramuscular sedation with dexmedetomidine (375 μg/m^2^ body surface, Dexdomitor, Orion Corporation, Espoo, Finland), an intravenous 22G over-the-needle catheter (Optiva, Jelco Smiths Medical International Ltd, Rossendale, UK) was placed in one of the cephalic veins. The animals were then allowed to relax in a quiet room until an appropriate level of sedation was obtained. The sedation scoring system, described by Carter et al. [39] was used. The level of sedation was assessed on a numeric rating scale where 0 = no sedation; 1 = standing or sitting and head lower than before sedation; 2 = standing or sitting unbalanced, muscle weakness and refusing to lie down; 3 = lying in sternal recumbency but responsive; 4 = lying in lateral recumbence but responsive; and 5 = lying in lateral recumbency and unresponsive. When a sedation score of 4 or 5 was reached, usually after 20 to 30 minutes, the infusions were started. A constant rate infusion (CRI) of ketamine or saline was given intravenously at a rate of 0.0125 ml/kg/min, over a 40-minute time period using a syringe driver (B. Braun Melsungen AG, Perfusor space, Melsungen, Germany). During the infusions, the principal investigator monitored the animals and cardiovascular parameters and sedation scores were evaluated every ten minutes. Following termination of the infusion, the cephalic catheter was removed and the animals were allowed to recover.

### Anaesthetic protocol

SPECT acquisitions were obtained under general anaesthesia. All dogs were premedicated intramuscular with 375 μg/m^2^ body surface dexmedetomidine approximately 30 minutes prior to induction in a quiet dimmed room. Next, a 22G over-the-needle cephalic catheter was placed in one of the front legs to gain intravenous access. General anaesthesia was induced with propofol (Propovet Multidose, Abbott Laboratories, Berkshire, United Kingdom) given intravenously to effect and maintained with isoflurane (Isoflo, Abbott Laboratories, Berkshire, UK) in oxygen using a rebreathing system. During the general anaesthesia, heart rate, respiratory rate, end tidal carbon dioxide concentration and arterial haemoglobin oxygen saturation were measured by means of a calibrated multigas analyzer (Capnomac Ultima, Datex, Helsinki, Finland) and a pulse oximeter (N5500 Patient Monitor, Nellcor Puritan Bennett Inc., Pleasanton, CA, U.S.A.).

### SPECT perfusion imaging

Regional cerebral blood flow was evaluated with SPECT using the ^99m^Tc-labelled tracer HMPAO (d, 1, hexamethylpropylene amine oxime, Ceretec, GE healthcare LTD, UK). This is a validated technique to study rCBF in dogs [40,41]. The lipophilic tracer passes the blood brain barrier and is taken up by neurons, in a manner proportionally to cerebral blood flow and neuronal function. In the neurons, it is transformed into a hydrophilic form and becomes trapped intracellular [40]. After dexmedetomidine premedication, a cephalic catheter was placed and the tracer was administered intravenously (30.9 MBq/kg ± 3.0 MBq). All SPECT acquisitions were started between 10 to 20 minutes after tracer injection. Prior to the acquisition, all dogs were placed in ventral recumbence. The triple headed gamma camera (Triad, Trionix, Twinsburg, OH, USA) was equipped with low-energy ultrahigh resolution parallel hole collimators (tomographic resolution full width at half maximum = 9 mm). To facilitate comparison between dogs, camera and table positioning were standardized. Data were acquired over a circular 360-degree rotation, for 20 minutes in step-and-shoot mode (120 steps, 10 seconds per step, 3 degree per step) on a 128 x 128 matrix. Images were then processed using iterative reconstruction and a Butterworth filter (cut-off 1.6 cycli/cm, order 10, ten iterations, eight subsets). Pixel size was 1.72 mm [24].

### Image analysis

**Fig 2:**
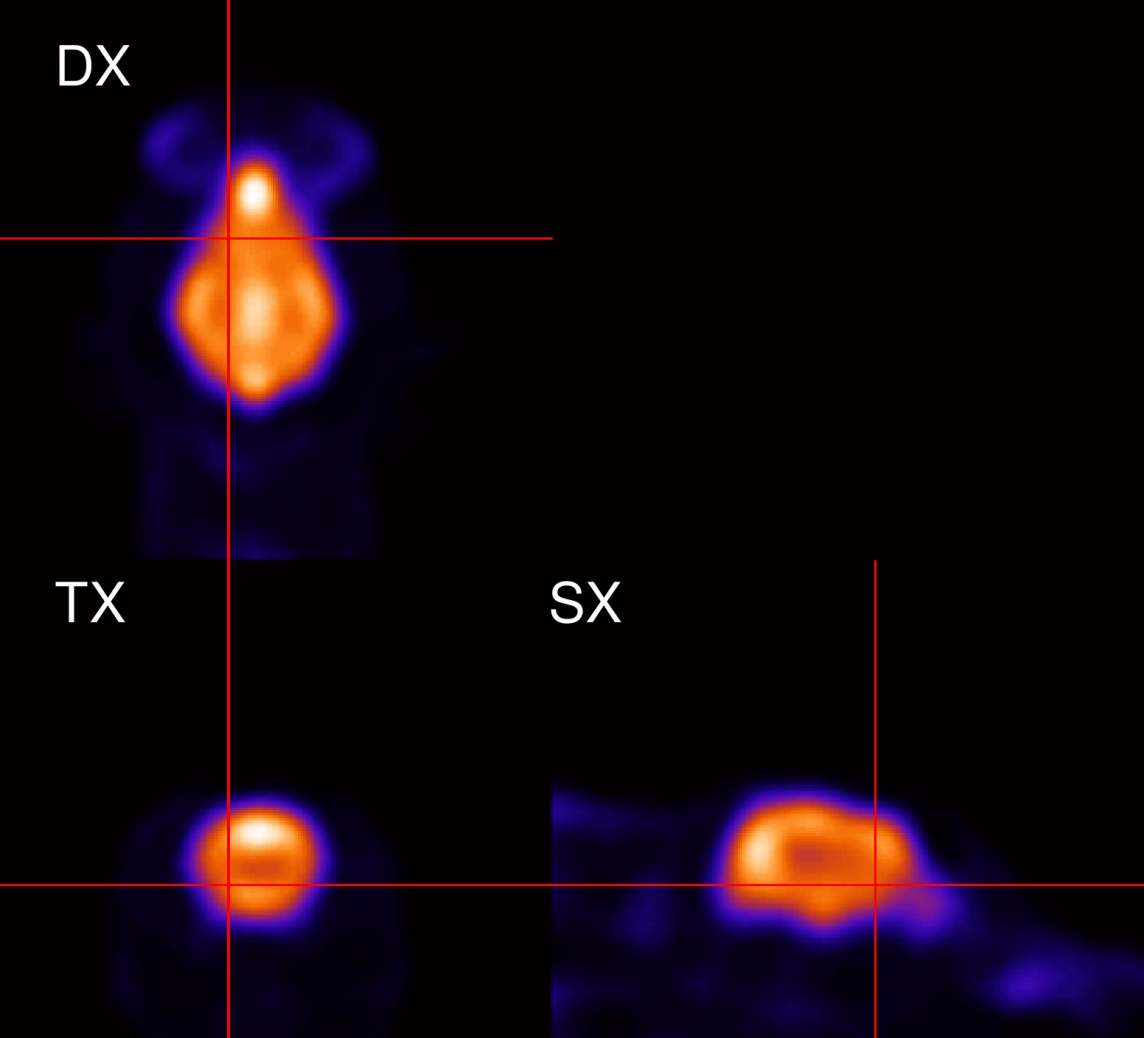
Dorsal (HX), transversal (TX) and sagittal (SX) images of the brain of a dog, obtained with ^99m^Tc-HMPAO SPECT. The left frontal cortex is indicated by the red cross.

The individual patient’s perfusion images were automatically registered to a template, generated from 14 dogs (9 males, 5 females, mean age 50 months ± 20), using Brain Registration and Automated SPET Semiquantification software (BRASS, Nuclear diagnostics, Sweden) [22]. This template based automated registration method eliminates subjective operator dependent region definition and the automatic registration (allowing shifting, scaling and rotation of the data) facilitates the fitting procedure to the template, necessary to compensate for intra-individual differences in anatomical brain size and shape. On this template, a region map was generated, including 11 separate manually drawn volumes of interest (VOI) positioned over the frontal, temporal, parietal and occipital lobes of both hemispheres as well as over the cerebellum and the basal ganglia and thalamus (Fig 2). To calculate the rCBF ratios (perfusion index (PI)), regional radioactivity was normalized to the radioactivity of the entire brain.

### Statistical analysis

A one-way repeated measure ANOVA was set up for each subanaesthetic ketamine dose for the left frontal cortex. The significance level was set at 0.05. The assumptions of normality of the error term, homoscedasticity and independence of the error term were checked by means of diagnostics plots and statistical tests (Shapiro Wilk, Levene’s Test). After Bonferroni correction for multiple testing, the remaining 10 brain regions were post hoc assessed with a type I error of 0.005. The analysis was performed in SPSS 23 (The Statistical Package for Social Science SPSS Inc, USA).

Paired student’s *t-*statistics were applied to compare pre and post ketamine C-BARQ scores. The significance level was again set at 0.05.

For the evaluation of the heart rate, statistical analysis was computed using Rstudio 1.0.136 (R: A Language and Environment for Statistical Computing; R Core Team; R Foundation for Statistical Computing, Vienna, Austria, 2016, https://www.R-project.org/) with packages MASS (version 7.3–45), doBy (version 4.6–1), lme4 (version 3.1–137) and LmerTest (version 3.0–1).

Onto the data set a nested linear mixed model with heterogeneous (unstructured) variances was set up. The model was written as E(Yt|T1,T2) = β0 + β1t1 + β2t2 + β3t3 + β4t4 + β5T1 + β6T2+ β7t1T1 + β8t2T1 + β9t3T1 + β10t4T1 + β11t1T2 + β12t2T2 + β13t3T2 + β14t4T2 with Yt as response variable. Heart rate was set as response value whereas time infusion and treatment (both categorical) were set as predictor value. The factors infusion and animal (categorical) were set as random factors. The predictor time infusion (t) denotes the different time points with t1 the first of five (k-1 = 5–1 = 4) dummies (= 1 if time point = “10 minutes” or 0 otherwise), t2 the second dummy (= 1 if time point = “20 minutes” or 0 otherwise), t3 (= 1 if time point = “30 minutes” or 0 otherwise) and t4 (= 1 if time point = “40 minutes” or 0 otherwise). The treatment predictor (T) indicated the different treatment modalities with T1 the first of two (k-1 = 3–1 = 2) dummies (= 1 if treatment = “0.5 mg/kg” or 0 otherwise) and T2 (= 1 if treatment = “2 mg/kg” or 0 otherwise). The reference level (for each region) was set as the heart rate at baseline in condition saline (intercept). The type-I error was set at 0.05 (two-tailed). The assumptions of linearity of the regression function and the normality of the error term were assessed by making diagnostics plots.

## Results

**Fig 3:**
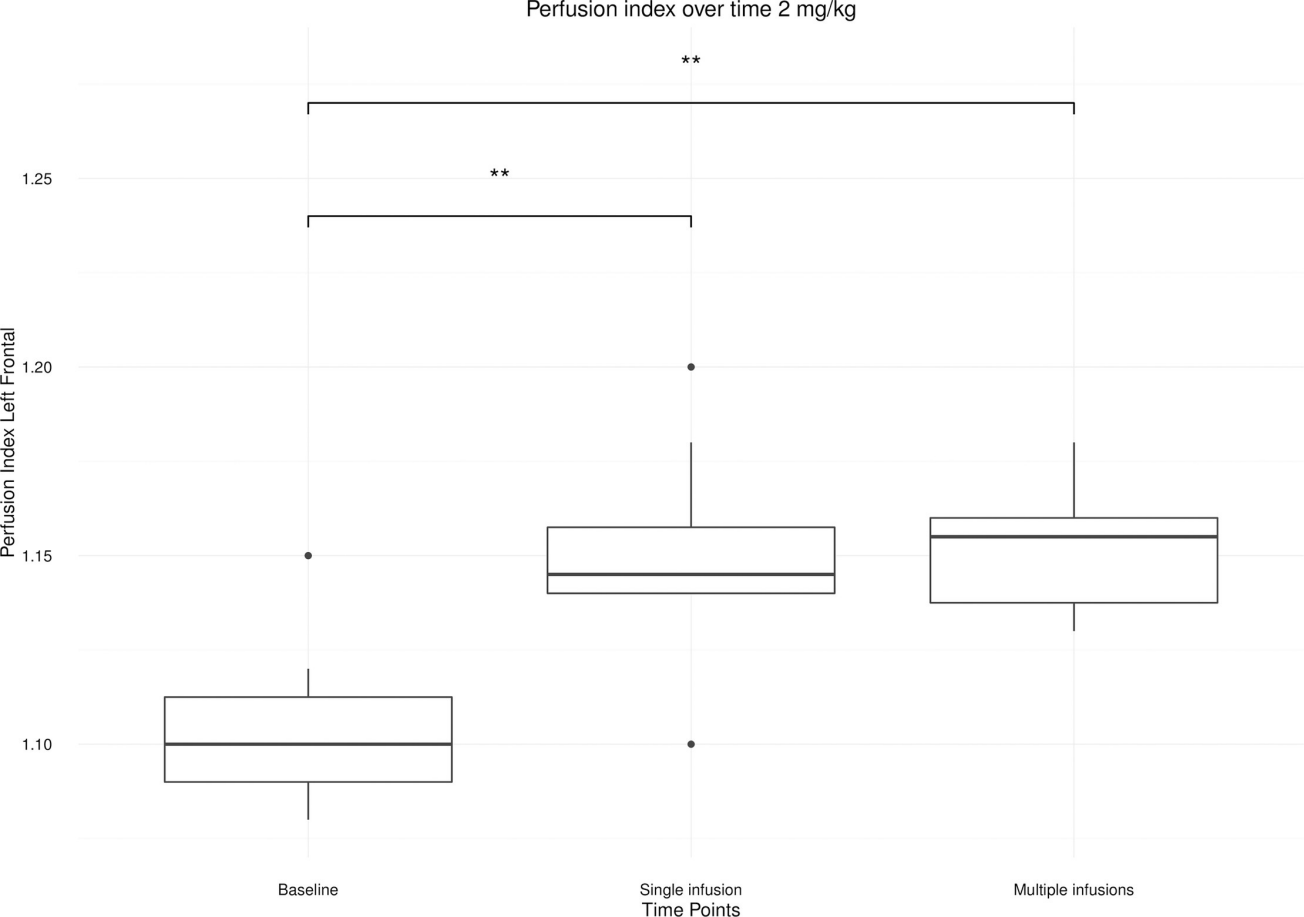
Boxplot of the left frontal cortex perfusion index after single and multiple ketamine infusions relative to baseline for the 2 mg/kg condition. (*< 0.01; **< 0.001).

Table 1 shows that the average PI of the saline and 0.5 mg/kg condition stays at baseline for the left frontal cortex. Within the 2 mg/kg condition the average PI of the left frontal cortex differed significantly over time (*p* = 0.003). This dose provoked in this region an increase in PI 24 hours after a single infusion (*p* = 0.001), which remained at that level 24 hours after completion of the infusion series (*p* = 0.001) (Fig 3).

**Table 1:** Descriptive statistics for the perfusion index of the left frontal cortex and thalamus, measured at baseline and 24 hours after single and multiple ketamine (0.5 or 2 mg/kg) or saline infusions (n = 24).

	Baseline				Single infusion				Multiple infusions			
	*Mean*	*SD*	*Min*	*Max*	*Mean*	*SD*	*Min*	*Max*	*Mean*	*SD*	*Min*	*Max*
**Left frontal**												
saline	1.12	0.04	1.05	1.18	1.13	0.03	1.08	1.17	1.14	0.02	1.11	1.16
0.5 mg/kg	1.12	0.02	1.07	1.14	1.13	0.03	1.08	1.19	1.12	0.02	1.08	1.15
2 mg/kg	1.11	0.02	1.08	1.15	1.15	0.03	1.10	1.20	1.15	0.02	1.13	1.18
**Thalamus**												
saline	1.17	0.07	1.02	1.25	1.17	0.05	1.10	1.25	1.17	0.03	1.12	1.22
0.5 mg/kg	1.18	0.05	1.10	1.27	1.19	0.06	1.10	1.27	1.18	0.06	1.10	1.25
2 mg/kg	1.15	0.05	1.06	1.24	1.18	0.04	1.12	1.25	1.19	0.05	1.15	1.31

**Fig 4:**
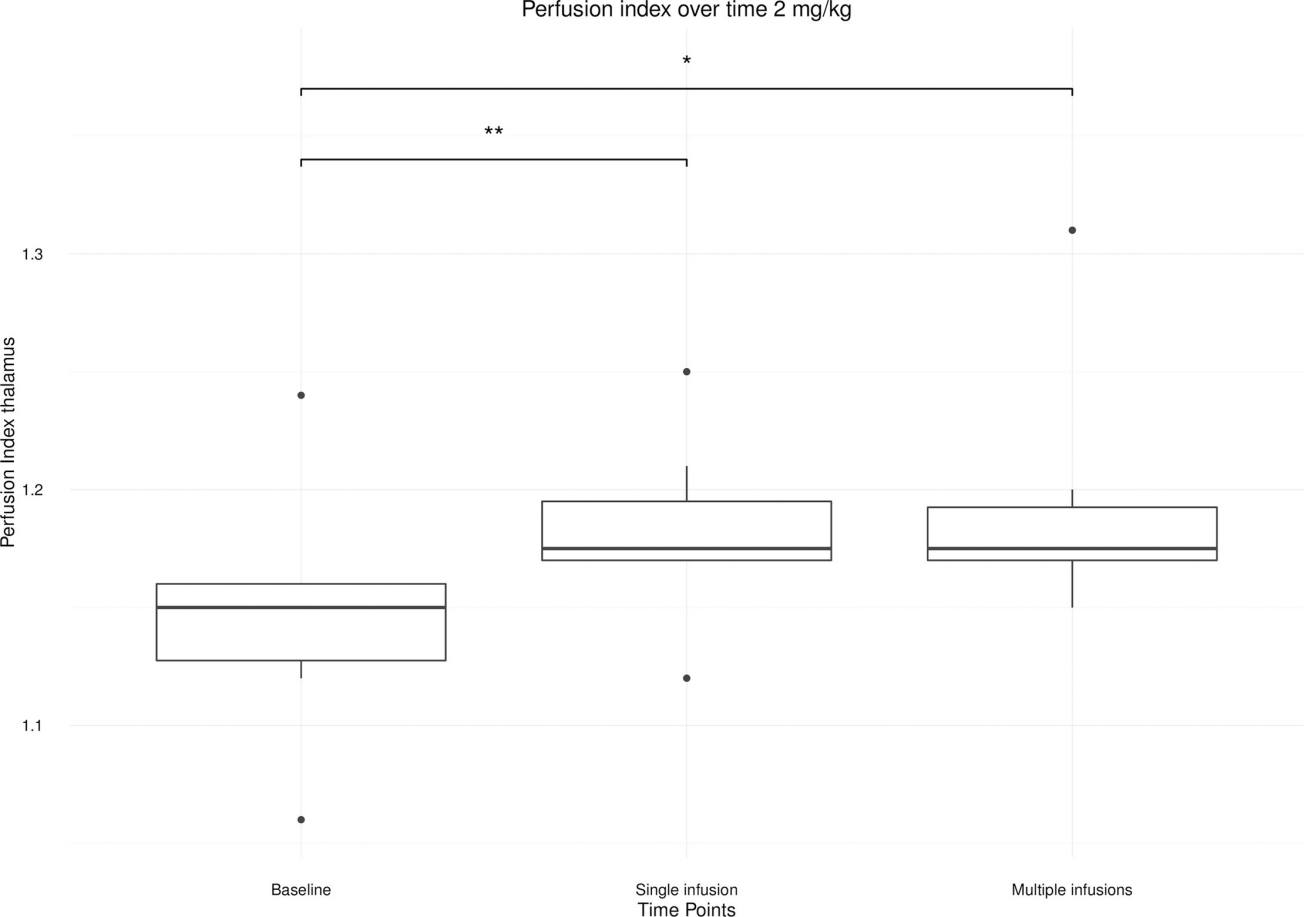
Boxplot of the perfusion index of the thalamus after single and multiple ketamine infusions relative to baseline for the 2 mg/kg condition. (*< 0.01; **< 0.001).

The post-hoc analysis revealed only a significant difference within the 2mg/kg condition for the thalamic region (*p* = 0.004). At this dose, perfusion index was significantly increased 24 hours after single (p = 0.001) and multiple (p = 0.006) ketamine infusions compared to baseline (Fig 4).

Concerning the heart rate, the linear mixed model revealed for the 0.5 mg/kg condition a significant time by treatment interaction for the time points 3 to 5 during the ketamine infusions (S2 Table). These results indicate an increase in heart rate at 20 (p = 0.003), 30 (p = 0.003) and 40 minutes (p = 0.015) after the start of the infusion, compared to the reference level. For the 2mg/kg condition, all time by treatment interaction terms were significant (with p < 0.001), indicating a significant increase in heart rate at 10, 20, 30, and 40 minutes after the start of the infusion (S2 Table). However, at all time points during the ketamine infusions, heart rate remained within physiological limits [42] and no clinically relevant adverse effects were observed.

In all three treatment conditions, the C-BARQ questionnaire revealed no significant differences in behaviour scores after ketamine infusions relative to baseline values.

## Discussion

In this healthy dog study, a single subanaesthetic dose of ketamine (2mg/kg) caused an increase in regional cerebral perfusion in the left frontal cortex and in the thalamic region. In the 0.5 mg/kg condition, as well as in condition saline, no significant changes in cerebral perfusion were observed. The perfusion alterations in the 2 mg/kg condition were still present 24 hours after ketamine infusion. Similar increases in left frontal blood flow were demonstrated in another SPECT study after acute subanaesthetic ketamine infusion in dogs [35]. Also in humans, several studies investigating the effect of subanaesthetic doses of ketamine have demonstrated increases in rCBF in frontal and thalamic regions [33,34,37]. These similar results in both species support the assumption to use the dog as a valuable animal model to investigate the neurobiological effects of low-dose ketamine infusions in humans. However, all of the studies mentioned above, both in dogs and humans, evaluated rCBF during or shortly after a single ketamine infusion. In contrast, our study showed that the regional changes in cerebral blood flow were still detectable 24 hours after ketamine administration corresponding with peak antidepressant responses observed in human patients [43]. These prolonged perfusion alterations, beyond ketamine’s biological half-life, are in line with ketamine’s sustained antidepressant actions in human psychiatric diseases and could be one of the mechanisms through which ketamine exerts its effects. Nevertheless, regional brain perfusion 24 hours after a single ketamine infusion did not differ from rCBF measured 24 hours after multiple infusions. This is in contrast with human literature on major depression, where it is described that repeated ketamine infusions are associated with higher response and remission percentages than single infusions [13–15]. However, in our study, only short term effects of single and repeated ketamine infusions were examined and therefore prolonged perfusion alterations following repeated ketamine administration cannot be excluded.

Significant perfusion changes were only detected in the 2 mg/kg condition. In human psychiatry, ketamine is generally used at a dose of 0.5 mg/kg, with evident clinical responses [6–9]. In addition, several perfusion studies showed that these low doses were able to increase rCBF in frontal and thalamic regions [33,34,37]. In our study, a higher dose was needed to provoke significant changes in cerebral perfusion in dogs. A possible explanation could be the difference in half-life of ketamine between both species. In dogs, the elimination half-life of intravenous ketamine is approximately 1 hour [18], whereas a half-life of approximately 3 hours is reported in humans [6,44]. Moreover, a higher dose is needed to induce anesthesia in dogs in comparison with humans [18,45].

The changes in brain perfusion observed after administration of subanaesthetic ketamine are in line with alterations in cerebral perfusion caused by several other antidepressant treatment options in humans. Decreased perfusion and hypoactivity of the frontal cortex and subcortical limbic structures (including the thalamus) are common characteristics in patients with MDD and anxiety disorders [26,27,29–31]. Furthermore, response to antidepressant therapy is generally associated with normalization of rCBF abnormalities in these brain regions. Increased perfusion in frontal and subcortical brain regions has been demonstrated in patients responding to treatment with selective serotonin reuptake inhibitors and tricyclic antidepressants [46–48]. Similar changes in brain perfusion were identified in patients successfully treated with high frequency repetitive transcranial magnetic stimulation over the left frontal cortex [49,50]. Less consistency is found regarding the changes in rCBF caused by electroconvulsive therapy (ECT) [46,51–53].

We observed profound lateral asymmetry in frontal cortical perfusion after subanaesthetic ketamine administration, with significant increases in perfusion solely noted in the left hemisphere. This is noteworthy given the fact that reductions in rCBF of the frontal cortex in MDD patients are predominantly observed on the left [54,55]. In line with rCBF abnormalities found in humans suffering from MDD and anxiety disorders, also in dogs with pathological anxiety lower left frontal and subcortical blood flow has been reported [24]. This is an interesting finding, as it means that ketamine could be a valuable adjunctive treatment for dogs with an incomplete response to standard behavioural therapy and pharmacotherapy, or for dogs with the need of a faster response. Indeed, additional treatment options for anxiety-disordered dogs are needed, since the management of behavioural disorders is challenging and treatment outcome is often unsatisfactory [56–59]. The observations made in the current study strengthen the hypothesis that ketamine’s influence on rCBF could be an explanation for its antidepressant effects and support the possible use of ketamine as an adjunctive treatment for anxiety in dogs.

Some authors suggest that the presence or the intensity of dissociative or psychotomimetic effects during ketamine infusion predicts a more robust and sustained antidepressant response [60–62]. Even more, the question has raised whether these effects are necessary for rapid antidepressant effects to occur. In this study, no adverse behavioural effects were observed during the ketamine infusions, due to the fact that the dogs were sedated with dexmedetomidine. Furthermore, using the C-BARQ questionnaire, no behavioural changes could be identified following the ketamine infusions. However, since this study included normal dogs, it remains to be explored whether dexmedetomidine blocks the psychotomimetic effects in behavioural disordered animals to the same extent.

Using the dexmedetomidine sedative protocol, no clinically relevant cardiovascular effects were observed during the ketamine infusions.

An important limitation of this study is the fact that the dogs were sedated with dexmedetomidine during the ketamine infusions. However, it seems unlikely that this would have influenced scan results, as all scans were performed 24 hours later and dexmedetomidine has a short half-life of approximately one hour [63]. Dexmedetomidine was administered to avoid side effects due to the ketamine administration. In human medicine, it was shown that dexmedetomidine reduced delirium, excitement, and hallucinations caused by ketamine and produced more haemodynamic stable patients [64]. Also in veterinary medicine, dexmedetomidine–ketamine is a frequently used combination which provides heavy sedation or general anaesthesia with less behavioural side effects than ketamine alone [65]. Potential adverse effects in dogs include sympathomimetic symptoms such as tachycardia and hypertension, excitement, increased muscle tone and hypersalivation [18]. In the current study, no behavioural or cardiovascular adverse effects were observed. Another limitation lies in the fact that the dogs were under general anaesthesia during the brain imaging. As a result, the sedatives and anaesthetics used could have influenced imaging results. However, the created images during scanning process are a reflection of the situation at the moment of tracer injection, as the tracer becomes trapped intracellular within minutes after injection. Consequently, all anaesthetics administered later on will have no influence on scan results. Still, sedation with dexmedetomidine was performed before tracer injection. However, the influence of this sedative on brain perfusion is established and was taken into account when interpreting results [36,66]. Important to notice is the fact that an intra-subject comparison was carried out, evaluating the same animal before and after ketamine administration with an identical anaesthetic protocol thereby eliminating potential sedation effects. In addition, the study design included a placebo group (condition saline) in order to further exclude sedation effects. A final limitation is that the clinical importance of the observed cerebral perfusion alterations could not be assessed in this study.

## Conclusions

In conclusion, this study demonstrates a dose-related increased regional cerebral blood flow in the left frontal cortex and thalamus 24 hours after single and multiple subanaesthetic ketamine administration in healthy dogs. These observations are especially interesting because the brain regions involved are important in canine and human mood disorders. Therefore, in a further study, it would be interesting to examine ketamine’s clinical effects in a population of anxiety-disordered dogs. In addition, the difference in long term effects between single and multiple ketamine infusions should be examined.

## Supporting information

S1 Table Descriptive statistics for the perfusion index of the right frontal cortex, temporal cortex, parietal cortex, occipital cortex, cerebellum and basal ganglia, measured at baseline and 24 hours after single and multiple ketamine (0.5 or 2 mg/kg) or saline infusions (n = 24). (DOCX)

S2 Table Results for the fixed factors of the nested linear mixed model for analysis of the heart rate at five different time points (0, 10, 20, 30 and 40 minutes after the start of the infusion) during the ketamine infusions. (*< 0.05; **< 0.001).
(DOCX)
