# Supplementary material for: The influence of subanaesthetic ketamine on regional cerebral blood flow in healthy dogs measured with 99mTc-HMPAO SPECT
Source: PLoS One. 2018 Dec 18;13(12):e0209316. doi: 10.1371/journal.pone.0209316 (PMC6298672; doi:10.1371/journal.pone.0209316)
Supplement: S1 Table — (DOCX) [file pone.0209316.s001.docx]

|  |  | | **Baseline** | | | | | | | | | | |  | | | **Single infusion** | | | | | | | | | | |  | | | **Multiple infusions** | | | | | | | | |  |
| --- | --- | --- | --- | --- | --- | --- | --- | --- | --- | --- | --- | --- | --- | --- | --- | --- | --- | --- | --- | --- | --- | --- | --- | --- | --- | --- | --- | --- | --- | --- | --- | --- | --- | --- | --- | --- | --- | --- | --- | --- |
|  | | *Mean* | | | *SD* | | | *Min* | | | *Max* | | | | *Mean* | | | | *SD* | | | *Min* | | | *Max* | | | | *Mean* | | | | *SD* | | | *Min* | | | *Max* |  |
| **Right frontal** | | |  |  | | |  | | |  | | |  | | | | |  | | |  | | |  | | |  | | | | |  | | |  | | |  | |  |
| placebo | | | 1.10 | | | 0.02 | | | 1.06 | | | 1.13 | | | | 1.11 | | | | 0.02 | | | 1.07 | | | 1.15 | | | | 1.12 | | | | 0.02 | | | 1.09 | | 1.14 |  |
| 0.5 mg/kg | | | 1.10 | | | 0.02 | | | 1.06 | | | 1.13 | | | | 1.10 | | | | 0.04 | | | 1.03 | | | 1.15 | | | | 1.09 | | | | 0.03 | | | 1.03 | | 1.13 |  |
| 2 mg/kg | | | 1.10 | | | 0.02 | | | 1.08 | | | 1.13 | | | | 1.11 | | | | 0.02 | | | 1.07 | | | 1.13 | | | | 1.11 | | | | 0.03 | | | 1.07 | | 1.15 |  |
| **Left temporal** | | |  | | |  | | |  | | |  | | | |  | | | |  | | |  | | |  | | | |  | | | |  | | |  | |  |  |
| placebo | | | 0.92 | | | 0.03 | | | 0.86 | | | 0.95 | | | | 0.91 | | | | 0.02 | | | 0.89 | | | 0.95 | | | | 0.91 | | | | 0.02 | | | 0.87 | | 0.92 |  |
| 0.5 mg/kg | | | 0.91 | | | 0.02 | | | 0.88 | | | 0.95 | | | | 0.91 | | | | 0.03 | | | 0.86 | | | 0.96 | | | | 0.92 | | | | 0.02 | | | 0.90 | | 0.96 |  |
| 2 mg/kg | | | 0.92 | | | 0.05 | | | 0.82 | | | 0.96 | | | | 0.93 | | | | 0.02 | | | 0.90 | | | 0.96 | | | | 0.90 | | | | 0.04 | | | 0.82 | | 0.94 |  |
| **Right temporal** | | |  | | |  | | |  | | |  | | | |  | | | |  | | |  | | |  | | | |  | | | |  | | |  | |  |  |
| placebo | | | 0.90 | | | 0.03 | | | 0.84 | | | 0.94 | | | | 0.88 | | | | 0.03 | | | 0.83 | | | 0.92 | | | | 0.88 | | | | 0.02 | | | 0.85 | | 0.92 |  |
| 0.5 mg/kg | | | 0.90 | | | 0.03 | | | 0.85 | | | 0.93 | | | | 0.90 | | | | 0.02 | | | 0.87 | | | 0.93 | | | | 0.90 | | | | 0.02 | | | 0.88 | | 0.93 |  |
| 2 mg/kg | | | 0.88 | | | 0.03 | | | 0.85 | | | 0.92 | | | | 0.89 | | | | 0.02 | | | 0.86 | | | 0.91 | | | | 0.86 | | | | 0.05 | | | 0.77 | | 0.90 |  |
| **Cerebellum** | | |  | | |  | | |  | | |  | | | |  | | | |  | | |  | | |  | | | |  | | | |  | | |  | |  |  |
| placebo | | | 1.09 | | | 0.04 | | | 1.01 | | | 1.15 | | | | 1.10 | | | | 0.03 | | | 1.05 | | | 1.15 | | | | 1.10 | | | | 0.03 | | | 1.06 | | 1.16 |  |
| 0.5 mg/kg | | | 1.12 | | | 0.05 | | | 1.02 | | | 1.18 | | | | 1.09 | | | | 0.06 | | | 1.01 | | | 1.20 | | | | 1.08 | | | | 0.04 | | | 1.01 | | 1.14 |  |
| 2 mg/kg | | | 1.09 | | | 0.08 | | | 1.00 | | | 1.24 | | | | 1.08 | | | | 0.02 | | | 1.04 | | | 1.10 | | | | 1.08 | | | | 0.02 | | | 1.04 | | 1.11 |  |
| **Left parietal** | | |  | | |  | | |  | | |  | | | |  | | | |  | | |  | | |  | | | |  | | | |  | | |  | |  |  |
| placebo | | | 1.18 | | | 0.03 | | | 1.13 | | | 1.22 | | | | 1.18 | | | | 0.05 | | | 1.09 | | | 1.25 | | | | 1.19 | | | | 0.03 | | | 1.13 | | 1.23 |  |
| 0.5 mg/kg | | | 1.16 | | | 0.02 | | | 1.13 | | | 1.20 | | | | 1.17 | | | | 0.04 | | | 1.11 | | | 1.23 | | | | 1.19 | | | | 0.03 | | | 1.14 | | 1.23 |  |
| 2 mg/kg | | | 1.18 | | | 0.03 | | | 1.14 | | | 1.24 | | | | 1.17 | | | | 0.02 | | | 1.14 | | | 1.20 | | | | 1.17 | | | | 0.03 | | | 1.13 | | 1.21 |  |
| **Right parietal** | | |  | | |  | | |  | | |  | | | |  | | | |  | | |  | | |  | | | |  | | | |  | | |  | |  |  |
| placebo | | | 1.16 | | | 0.04 | | | 1.10 | | | 1.20 | | | | 1.16 | | | | 0.05 | | | 1.09 | | | 1.24 | | | | 1.15 | | | | 0.04 | | | 1.10 | | 1.21 |  |
| 0.5 mg/kg | | | 1.15 | | | 0.04 | | | 1.10 | | | 1.20 | | | | 1.14 | | | | 0.03 | | | 1.09 | | | 1.19 | | | | 1.16 | | | | 0.02 | | | 1.14 | | 1.21 |  |
| 2 mg/kg | | | 1.14 | | | 0.02 | | | 1.11 | | | 1.18 | | | | 1.14 | | | | 0.04 | | | 1.10 | | | 1.21 | | | | 1.14 | | | | 0.05 | | | 1.08 | | 1.21 |  |
| **Right occipital** | | |  | | |  | | |  | | |  | | | |  | | | |  | | |  | | |  | | | |  | | | |  | | |  | |  |  |
| placebo | | | 1.15 | | | 0.02 | | | 1.11 | | | 1.17 | | | | 1.15 | | | | 0.04 | | | 1.09 | | | 1.20 | | | | 1.12 | | | | 0.03 | | | 1.09 | | 1.14 |  |
| 0.5 mg/kg | | | 1.15 | | | 0.05 | | | 1.07 | | | 1.22 | | | | 1.14 | | | | 0.04 | | | 1.07 | | | 1.19 | | | | 1.15 | | | | 0.05 | | | 1.07 | | 1.24 |  |
| 2 mg/kg | | | 1.16 | | | 0.02 | | | 1.14 | | | 1.19 | | | | 1.13 | | | | 0.02 | | | 1.10 | | | 1.17 | | | | 1.14 | | | | 0.04 | | | 1.09 | | 1.20 |  |
| **Left occipital** | | |  | | |  | | |  | | |  | | | |  | | | |  | | |  | | |  | | | |  | | | |  | | |  | |  |  |
| placebo | | | 1.17 | | | 0.03 | | | 1.13 | | | 1.21 | | | | 1.18 | | | | 0.05 | | | 1.10 | | | 1.23 | | | | 1.14 | | | | 0.04 | | | 1.09 | | 1.19 |  |
| 0.5 mg/kg | | | 1.17 | | | 0.05 | | | 1.11 | | | 1.25 | | | | 1.15 | | | | 0.03 | | | 1.10 | | | 1.21 | | | | 1.16 | | | | 0.04 | | | 1.11 | | 1.22 |  |
| 2 mg/kg | | | 1.18 | | | 0.04 | | | 1.12 | | | 1.26 | | | | 1.15 | | | | 0.02 | | | 1.11 | | | 1.18 | | | | 1.18 | | | | 0.04 | | | 1.11 | | 1.23 |  |
| **Basal ganglia**  Placebo  0.5 mg/kg  2 mg/kg | | | 1.11  1.14  1.10 | | | 0.07  0.08  0.06 | | | 0.99  1.02  1.01 | | | 1.20  1.25  1.17 | | | | 1.10  1.13  1.17 | | | | 0.08  0.06  0.04 | | | 1.00  1.06  1.12 | | | 1.20  1.21  1.24 | | | | 1.15  1.13  1.21 | | | | 0.08  0.07  0.06 | | | 1.04  1.04  1.13 | | 1.25  1.21  1.29 |  |

**S1 Table. Descriptive statistics for the perfusion index of the right frontal cortex, temporal cortex, parietal cortex, occipital cortex, cerebellum and basal ganglia, measured at baseline and 24 hours after single and multiple ketamine (0.5 or 2 mg/kg) or saline infusions (n=24).**
