# Supplementary material for: The influence of subanaesthetic ketamine on regional cerebral blood flow in healthy dogs measured with 99mTc-HMPAO SPECT
Source: PLoS One. 2018 Dec 18;13(12):e0209316. doi: 10.1371/journal.pone.0209316 (PMC6298672; doi:10.1371/journal.pone.0209316)
Supplement: S2 Table — (*< 0.05; **< 0.001). (DOCX) [file pone.0209316.s002.docx]

|  | *Estimate* | *Standard error* | *p-value* |
| --- | --- | --- | --- |
| **(Intercept)**  **10 minutes**  **20 minutes**  **30 minutes**  **40 minutes**  **0.5 mg/kg**  **2 mg/kg**  **10 minutes :0.5 mg/kg**  **20 minutes :0.5 mg/kg**  **30 minutes :0.5 mg/kg**  **40 minutes :0.5 mg/kg**  **10 minutes :2 mg/kg**  **20 minutes :2 mg/kg**  **30 minutes :2 mg/kg**  **40 minutes :2 mg/kg** | 42.500  -0.200  -1.200  -0.400  1.100  -0.500  1.000  1.300  5.200  5.100  4.200  12.500  20.200  17.400  15.600 | 2.156  1.213  1.213  1.213  1.213  3.049  3.049  1.715  1.715  1.715  1.715  1.715  1.715  1.715  1.715 | <0.001**  0.869  0.323  0.742  0.365  0.871  0.745  0.449  0.003*  0.003*  0.015*  <0.001**  <0.001**  <0.001**  <0.001** |

**S2 Table. Results for the fixed factors of the nested linear mixed model for analysis of the heart rate at five different time points (0, 10, 20, 30 and 40 minutes after the start of the infusion) during the ketamine infusions.** (*< 0.05; **< 0.001).
